# Sample entropy analysis of cervical neoplasia gene-expression signatures

**DOI:** 10.1186/1471-2105-10-66

**Published:** 2009-02-20

**Authors:** Shaleen K Botting, Jerome P Trzeciakowski, Michelle F Benoit, Salama A Salama, Concepcion R Diaz-Arrastia

**Affiliations:** 1 Department of Obstetrics & Gynecology, University of Texas Medical Branch, Galveston, Texas, USA; 2 Northwest Medical Specialties, PLLC, Tacoma, Washington, USA; 3 Systems Biology and Translational Medicine, Texas A&M University Health Science Center, College Station, Texas, USA

## Abstract

**Background:**

We introduce Approximate Entropy as a mathematical method of analysis for microarray data. Approximate entropy is applied here as a method to classify the complex gene expression patterns resultant of a clinical sample set. Since Entropy is a measure of disorder in a system, we believe that by choosing genes which display minimum entropy in normal controls and maximum entropy in the cancerous sample set we will be able to distinguish those genes which display the greatest variability in the cancerous set. Here we describe a method of utilizing Approximate Sample Entropy (ApSE) analysis to identify genes of interest with the highest probability of producing an accurate, predictive, classification model from our data set.

**Results:**

In the development of a diagnostic gene-expression profile for cervical intraepithelial neoplasia (CIN) and squamous cell carcinoma of the cervix, we identified 208 genes which are unchanging in all normal tissue samples, yet exhibit a random pattern indicative of the genetic instability and heterogeneity of malignant cells. This may be measured in terms of the ApSE when compared to normal tissue. We have validated 10 of these genes on 10 Normal and 20 cancer and CIN3 samples. We report that the predictive value of the sample entropy calculation for these 10 genes of interest is promising (75% sensitivity, 80% specificity for prediction of cervical cancer over CIN3).

**Conclusion:**

The success of the Approximate Sample Entropy approach in discerning alterations in complexity from biological system with such relatively small sample set, and extracting biologically relevant genes of interest hold great promise.

## Background

Genomic heterogeneity is a characteristic feature of nearly all solid tumors, appearing early in tumor progression. Over time, early genomic instability evolutionarily leads to a molecularly heterogeneous population of cells naturally selected for their abilities to proliferate and invade, while simultaneously evading host defenses. The selection process, influenced by the unique to the tumor host environment, results in a further diverse intrapatient tumor population which is responsible for the clinical heterogeneity of the disease.

Concepts of tumorigenesis include the stochastic model, disputing the existence of genomic destabilization in tumor cells, instead arguing that mutations occurring during differentiation, development, and proliferation combine to create a cell with the perquisites for malignancy; according to this model, the cell then simply proliferates to create the cancer.[1] On the other end of the spectrum, the nature of tumor progression is viewed as a well-defined linear process paralleling normal cellular differentiation but accelerated in its steps due to acquired genomic instability producing multiple cell populations.[2] While each model has a degree of validity, solid tumors represent a far more chaotic process with diverse routes to genomic destabilization.

The evidence for genomic heterogeneity has been found in the extensive, progressive, and diverse genomic damage observed within the tumor, pre-neoplastic lesion, [3-5] and tumor-derived tissue culture cell lines.[6,7] If extensive genetic damage occurs or an essential gene target is effected, cell death will follow. However, the damage is often more subtle, without adverse consequences on cellular survival and no discernible significance. But those which promote proliferation, particularly by activating processes in the pathways of signal responsive cell proliferation or affecting removal of inappropriate proliferative cell populations, will give rise to expanding colonies harboring the mutant regulatory genes. Initially seen as an insignificant mass of cells, within this ever-increasing population of cells there exists a vast diversity of genomic damage out of which new advantageous mutations will be selected for processes such as adhesion, proteolysis, migration, lymphangiogenesis/angiogenesis, and immune escape mechanisms.

While a fortuitous random process is created by genomic destabilization and natural selection, most are searching for a coherent pattern within this extremely diverse event. It is likely that the observed clinical heterogeneity of cervical cancer is a reflection of the dissimilar genomic events between the patients. Since the advent of microarray technology, data generated from these experiments have most often been analyzed with the aim to identify specific patterns in the expression levels, such as periodicity or monotonous increases or decreases. [8-11] These methods of analysis, by their nature, exclude all genes which reflect an ill-defined pattern of expression within defined groups.

We introduce Approximate Sample Entropy as a mathematical method of analysis for microarray data. Approximate entropy is traditional utilized in analysis of temporal patterns; such as heart rate variability.[12,13] The utilitarian nature of entropy is beginning to be recognized in Microarray data analysis. Lezon et. al. recently applied entropy as a means identify metabolic oscillation within a cultured system presumably associated to the interaction of key genetic networks.[14] It is applied here as a method to classify the complex gene expression patterns resultant of a clinical sample set. Since Entropy is a measure of disorder in a system, we believe that by choosing genes which display minimum entropy in normal controls and maximum entropy in the cancerous sample set we will be able to distinguish those genes which display the greatest variability in the cancerous set. These genes can then be used to classify an unknown biopsy into one of these groups based on the calculated entropy of the sample.

Cervical cancer is an excellent model for evaluation of this analysis for multiple reasons. Aside from being the second leading cancer among women worldwide; with 470,000 new cases occurring annually; cervical cancer uniquely has an easily accessible, well defined, distinct pre-neoplastic lesion.[15]. The progression of disease to cancer is a linear process beginning with High-risk human papillomavirus (HPV) infection to intraepithelial neoplasia to invasive cancer. Squamous cell carcinoma of the cervix originates from a single carcinogenic trigger (HPV), yet as the lesion grows it develops to a heterogeneous population of cells characteristic of all solid tumors.

## Methods

### Sample handling and RNA isolation

Cervical biopsy samples were obtained from radical hysterectomy specimens of patients with Stage 1B1 squamous cell carcinoma of the cervix. Lesional and perilesional samples were obtained from 4 individual patients. Control biopsy samples were obtained from hysterectomy specimens of patients with documented normal cervix. All biopsies were histologically confirmed. Perilesional biopsies were normal cervical epithelium adjacent to the tumors.

Cervical biopsy samples from an additional 20 patients were utilized for real-time pcr validation. These biopsies were obtained from patients with either the preneoplasitc lesion (CIN 3), or with Stage 1A-1B squamous cell carcinoma of the cervix. As for the training set, normal cervical samples obtained from hysterectomy specimens of patients were utilized as controls. State of disease was histologically confirmed on an adjacent biopsy.

Total RNA was extracted from each sample using the RNAqueous RNA Isolation Kit (Ambion, Austin, Tx) following manufacturer's instructions. The extracted RNA is DNase treated prior to quantification. The RNA quality was assessed by detecting the 28S/18S peaks with an Agilent Bioanalyzer 2100 (Agilent Technologies, Walbronn, Germany). Only RNA with the highest fidelity (A260/A280 ratio between 1.9 and 2.1, and a high quality electropherogram) is used for microarray analysis

### Microarray analysis

Twenty μg of DNA-free RNA from each biopsy was applied to the GeneChip Human HG_U133 A & B GeneChip (Affymetrix). Microarray gene chip analysis was performed on all samples using the Affymetrix HU 133 A & B human 33,000-GeneChip (Affymetrix,) for identification of disease specific expression patterns. Labeling of cRNA, hybridization, and scanning of the microarrays were done according to the manufactures protocols (Affymetrix, Santa Clara, Calif.).

### Quantitative reverse transcription-PCR

Quantitative reverse transcription-PCR (qRT-PCR) was performed to validate differential expression of genes in an independent set of 20 cervical tissue biopsies. QRT-PCR was done with a 7500 Fast Real-time PCR system (Applied Biosystems) using gene-specific primer/probe mix (Assays-by-Design: Applied Biosystems, Foster City, Ca. The comparative threshold cycle (Ct) method was used in comparing the level of mRNA expression of the genes of interest to that of the internal control gene, GAPDH. Each reaction was set up in triplicate, with final expression levels in cancer samples compared to non-diseased, normal controls.

## Results

### Cluster Identification

**Figure 1:**
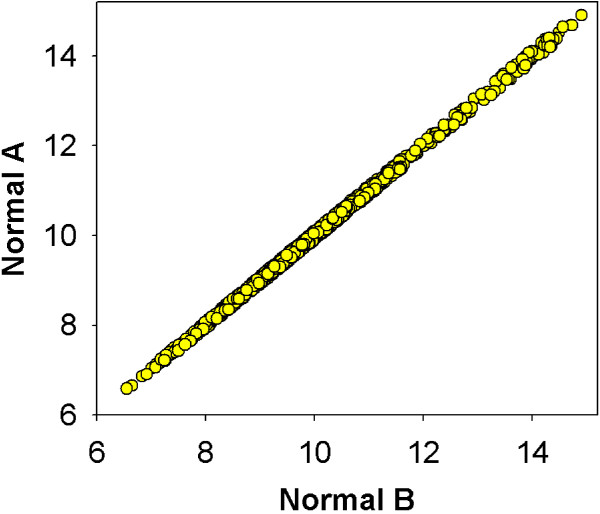
**Establishment of normal baseline for sample entropy calculations**. Initially a normal baseline was established, 966 genes were found to be unchanging between the normal samples when limited to a variance of less then 0.5% of the log2 expression values, a variance below the technical noise level of the Affymetrix array.

Initially a normal baseline was established, 966 genes were found to be unchanging between patients with normal cervical biopsies when limited to a variance of less then 0.5% of the log2 expression values, a variance below the technical noise level of the Affymetrix array.[16] (Figure 1)

Half of the normal patient's biopsies were used in the reference sample set while the other half was treated as experimental samples similar to the cancer and perilesional biopsy samples. Changes in gene expression were calculated for each of the 966 genes as

(1) ΔE = log_2 _(*reference*) - log_2 _(*experimental*) (1)

where *experimental *denotes expression in either the non-reference normal, perilesional or cancer samples. For use during the clustering phase, the Δ*E *values were averaged over subjects within each classification group (normal, perilesional, cancer) so that for any gene *k*, the mean Δ*E *is a 3-element vector

(2) $$\Delta {Ε}_{k}=\left(\Delta {Ε}_{k}^{1},\Delta {Ε}_{k}^{2},\Delta {Ε}_{k}^{3}\right)$$

with superscripts 1, 2, and 3 representing normal, perilesional and cancer sample groups, respectively.

The 3 by 966 array of mean Δ*E *values were input into a Bayesian classification algorithm (Ahlea Systems Corp., Nepean, ON) in MATLAB (MathWorks, Natick, MA) and the 966 genes were partitioned into subsets (classes) based on expression change patterns. The clustering partitioned the data in 3-space, where each dimension corresponded to one of the sample groups types. Genes were assigned to the cluster with the smallest Bayesian distance

(3) ΔE_*k *_∈ ω_*i *_if *d*_*i*_(Δ E_*k*_) <*d*_*j*_(ΔE_*k*_)   ∀ _*j *_≠ *i*

where the distances *d *were calculated as

(4) $$d_{i}(\Delta Ε)=\frac{1}{2}{\left(\Delta Ε-{\hat{\mu }}_{i}\right)}^{T}C_{i}^{-1}\left(\Delta Ε-{\hat{\mu }}_{i}\right)-\log\left[p\left(\omega_{i}\right)\right]+\frac{1}{2}\log\left[\left|C_{i}\right|\right]$$

${\hat{\mu }}_{i}$ and *C*_*i *_are the estimated mean and covariance matrix for cluster *ω*_*i *_and *p*(*ω*_*i*_) is the probability of membership in cluster *ω*_*i *_and |*C*_*i *_| is the determinant of the covariance matrix.

On the first pass through the clustering algorithm, all points were considered to be members of a single cluster located at a center given by the mean of each group over all 966 genes with an *a-priori *(or prior) probability *p*(*ω*_*i*_) of 1. On subsequent iterations, this quantity was based on the fraction of genes in each potential cluster.

The number of subsets was determined using a process of blind clustering which estimated the χ^2 ^statistic of hypothetical clusters to determine if the data set represents a single or multiple Gaussian distributions. If the computed chi^2 ^value for any cluster exceeded a set threshold (1.0 for 1 to 10 clusters; 2.0 for > 10 clusters) and if the product of the number of members of a class and the amount by which the chi-squared test exceeded the threshold was greater than a second criterion, the data were considered for further subsets. Subdivision of existing clusters continued until these conditions were no longer met. By varying the second criterion, it is possible to stabilize at a greater or lesser total number of clusters.

**Figure 2:**
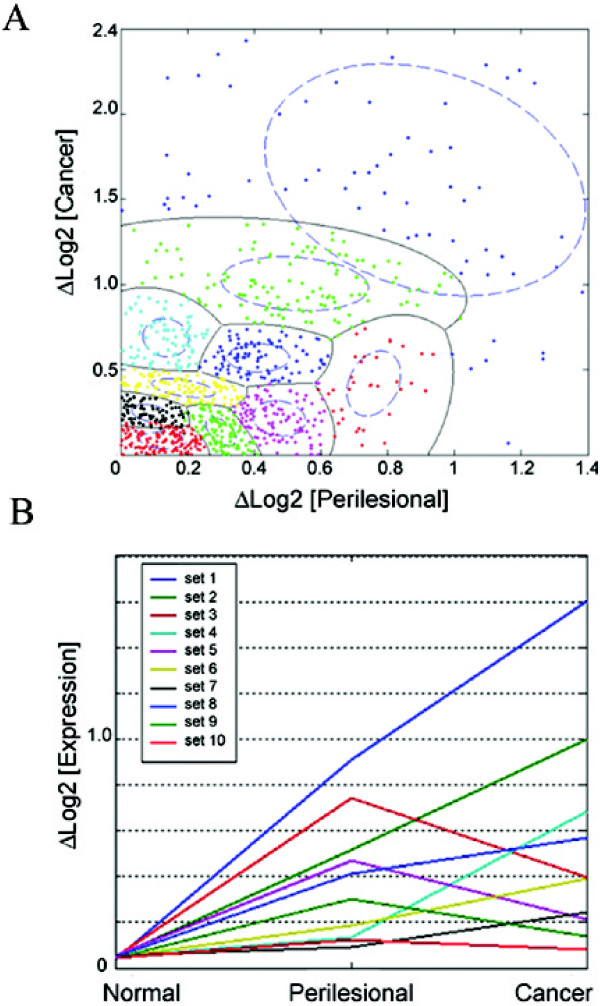
**Bayesian classification algorithm partitioned the genes into 10 subsets based on expression change patterns**. Scatter plot of the change in log2 expression of Cancer from Normal, versus Perilesional from Normal, depicts the 10 possible subsets of genes differentiating the two groups. Subsets 1 and 2 displayed the best predictive value during validation.(A) Line plot of log2 expression of the 10 subsets, for the average change of Log2 expression of from Normal; for Normal, Perilesional and Cancer for each subset.(B).

If an existing cluster was considered for further subdivision, two new cluster centers were located symmetrically along the principal axis (eigenvector) by a distance determined by the magnitude of the principal component (eigenvalue) computed from that cluster's covariance matrix. Bayesian distances were then estimated relative to the new clusters and the process was repeated. (Figure 2)

### Apparent Entropy Calculation

**Figure 3:**
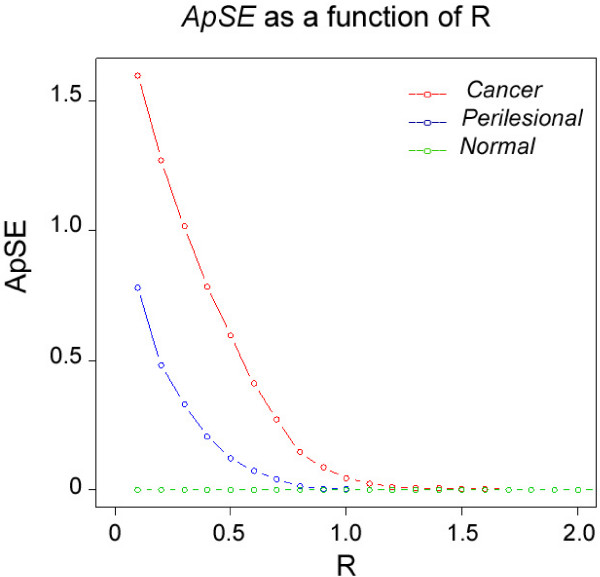
**ApSE as a function of R**. *ApSE *is calculated at 55 values of *R *ranging from 0.1 to 5.5 (only the first 20 points are plotted here). This figure depicts the *ApSE *values in a training set, where red, blue and green curves correspond to data from cancer, perilesional and normal biopsy samples. For simplicity, only the mean curve for each group is shown here. For prediction purposes, a separate *ApSE *curve is computed for each subject. Because of the between subjects variation in the *ApSE *curves, selecting optimal decision criteria by eye based on data for all subjects is not possible. Selection of classification rules in training sets and application of the rules in validation sets were therefore carried out using classification trees.

After dividing the 966 genes into 10 groups based on cluster membership, *ApSE *values were computed for the genes within each cluster. (Figure 3) The entropy of a sample may be approximated *(ApSE) *as the negative logarithm of the conditional probability *P(x) *that a dataset of sample *x*, falling within a tolerance range *(R) *for point *k*, will also repeat itself for each point in the set. Suppose | (Δ*E*)_*n *_| represents the absolute value of Δ*E *for subject *n *belonging to one of the three biopsy groups. Let *S*_*R *_denote the set of genes whose Δ*E *value for that subject falls within a neighborhood *R *of zero:

(5) *S*_*R *_= {*k*: (ΔE_*k*_)_*n *_<*R*}

where *R *is a real number ranging from 0.1 to 5.5 in steps of 0.1.

We can then define *A(R) *as the number of genes in set *S*_*R*_. If *N *is the total number of genes in the cluster being considered, then the approximate sample entropy can be written as:[17]

(6) $$ApSE\left(R\right)=-\log_{2}\frac{1+A\left(R\right)}{1+N}$$

### Classification Trees

Classification consisted of two stages: 1) determination of decision rules based on the *ApSE *scores and known biopsy classes of the subjects in the training set and 2) application of the decision rules to the *ApSE *values of subjects in the validation set to predict biopsy class. The agreement between the actual and predicted biopsy class was tabulated and scored.

Decision rules were determined separately for each subset of genes. ApSE values from the training set were input as independent/explanatory variables into a regression tree (Insightful Miner V3.01, Insightful Corp., Seattle WA). (Although Insightful Miner uses the term 'regression tree', the same process can be used to predict categorical or continuous dependent/response variables. The result is typically termed a regression tree when the response variable is continuous and a classification tree when the response variable is categorical).

The tree-fitting algorithm used in Insightful Miner is based on the recursive partitioning code called RPART.[18] Similar tree-fitting routines are available for R  in the downloadable "tree" library. As with the "tree" routine in S-Plus, this methodology builds upon the CART model developed by Breitman et al.. [19] A summary of the algorithms used in tree-based methods may be found in Venables and Ripley.[20] A comprehensive survey with proofs and theoretical results is given by Ripley.[21]

**Figure 4:**
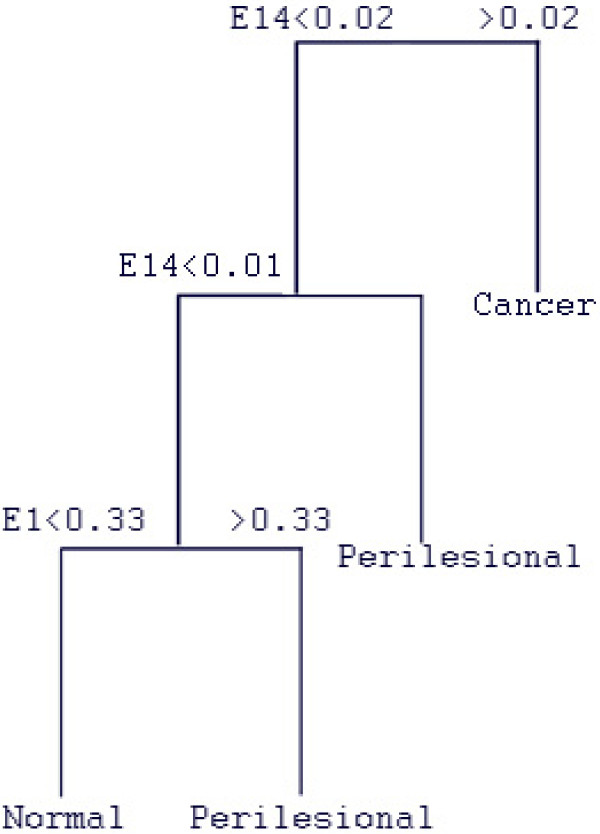
**Example Classification Tree based on *ApSE *analysis for a training set**. For this particular gene cluster, key decisions were based on the values of *ApSE *at R = 0.1 (E1) and at R = 1.4 (E14). All subjects whose *ApSE *value at R = 1.4 was greater than 0.02 were classed as belonging to the cancer group. For subjects with an *ApSE *value at R = 1.4 less than 0.02 were further split into two groups. If the *ApSE *at R = 1.4 was above 0.01, they were assigned to the perilesional group. In the subset of subjects whose *ApSE *at R = 1.4 was less than 0.01, the *ApSE *at R = 0.1 was used to make the final decision: subjects whose *ApSE *at R = 0.1 fell below 0.33 were classed as belonging to the normal group, with the remainder (*ApSE *at R = 0.1 > 0.33) falling into the perilesional group. These decision rules were then applied to a corresponding validation set of subjects (for the same cluster of genes) to predict the biopsy class.

Unlike traditional regression techniques which use a weighted sum of independent values to estimate the dependent variable, regression trees output a hierarchical series of rules or logical if-then conditions that can be used to classify the cases based on the values of the predictor variables.(Figure 4) Tree-based methods are well-suited for these kinds of classifications as they make no implicit assumptions regarding the distribution of or relationships between the predictor and dependent variables. In the final (cross-validation) stage of this analysis, the decision rules obtained using the training data were applied to the validation data set, and the agreement between the actual and predicted biopsy class was tabulated for each subset of genes.

### Quantitative real-time PCR validation of gene subsets

Ten genes were chosen for initial quantitative real-time validation of the gene array, for the two gene subsets (subset 1 and 2) which best represent our progressive model of disease. RNA was extracted from fresh frozen biopsies of normal, cervical intraepithelial neoplasia grade 3 (CIN 3) and stage IB-IIB squamous cell carcinoma (SCC) for Quantitative Real-Time PCR (qRT-PCR) using the RNAqueous RNA Isolation Kit (Ambion, Austin, Texas). Using the 7500 FAST Real-Time System (Applied Biosystems), cDNA from the samples was amplified in a single-plex reaction, for the following genes of interest (Adaptor related protein complex 2, sigma 1 subunit (AP2S1), Collagen typeVI alpha 3(Col6A3), Fibronectin 1 (FN1), Growth arrest specific 6 (GAS6), Human leukocyte antigen (HLA-C), High-mobility group nucleosome binding domain 1 (HMGN1), Heat shock 60 kDa protein 1 (HSPD1), Interferon gamma-inducible protein 16 (IFI16), Matrix metallopeptidase 2 (MMP2), Proteasome subunit beta-2 PSMB2, Tenascin C (TNC))

## Discussion

We believe that the observed clinical heterogeneity of most cancers is a reflection of the gross genomic heterogeneity and subsequently the molecular heterogeneity also found within the tumors, also that the more heterogeneous the cell population, the more likely the development of a metastatic, aggressive phenotype. Genomic destabilization, evolution, and selection for invasive, proliferating populations of cells encompass the fundamental nature of cancer. Cancer cells represent a diversely complex evolutionary life within the host. The resultant heterogeneous tumor is linked to its genetic state and represented in its gene expression profile. We have shown here that encompassing this heterogeneity in the data analysis aids in capturing the diversity within the cancer subgroups and in the identification of subgroup specific gene expression profiles.

Since the advent of microarray technology, data generated from these experiments have most often been analyzed with the aim to identify specific patterns in the expression levels, such as periodicity or monotonous increases or decreases. [8-11] These methods of analysis, by their nature, exclude all genes which reflect an ill-defined pattern of expression within defined groups. It is within these complex gene expression patterns that we find evidence for the extreme heterogeneity created by the genomic destabilization and natural selection processes that drives the cancer.

Scientists have struggled with theoretical means to detect alterations in system complexities. Entropy, a measure of disorder or randomness has been used to classify complex systems externally viewed as chaotic. A limited number of studies have utilized the concept of entropy in gene expression analysis; all to enhance clustering techniques by minimizing entropy and thus the amount of disorder in the groups. [22-24] Conversely, we have established a classification method based on Approximate Sample Entropy as a measure of molecular heterogeneity within our system indicative of clinical behavior of the tumor.

**Figure 5:**
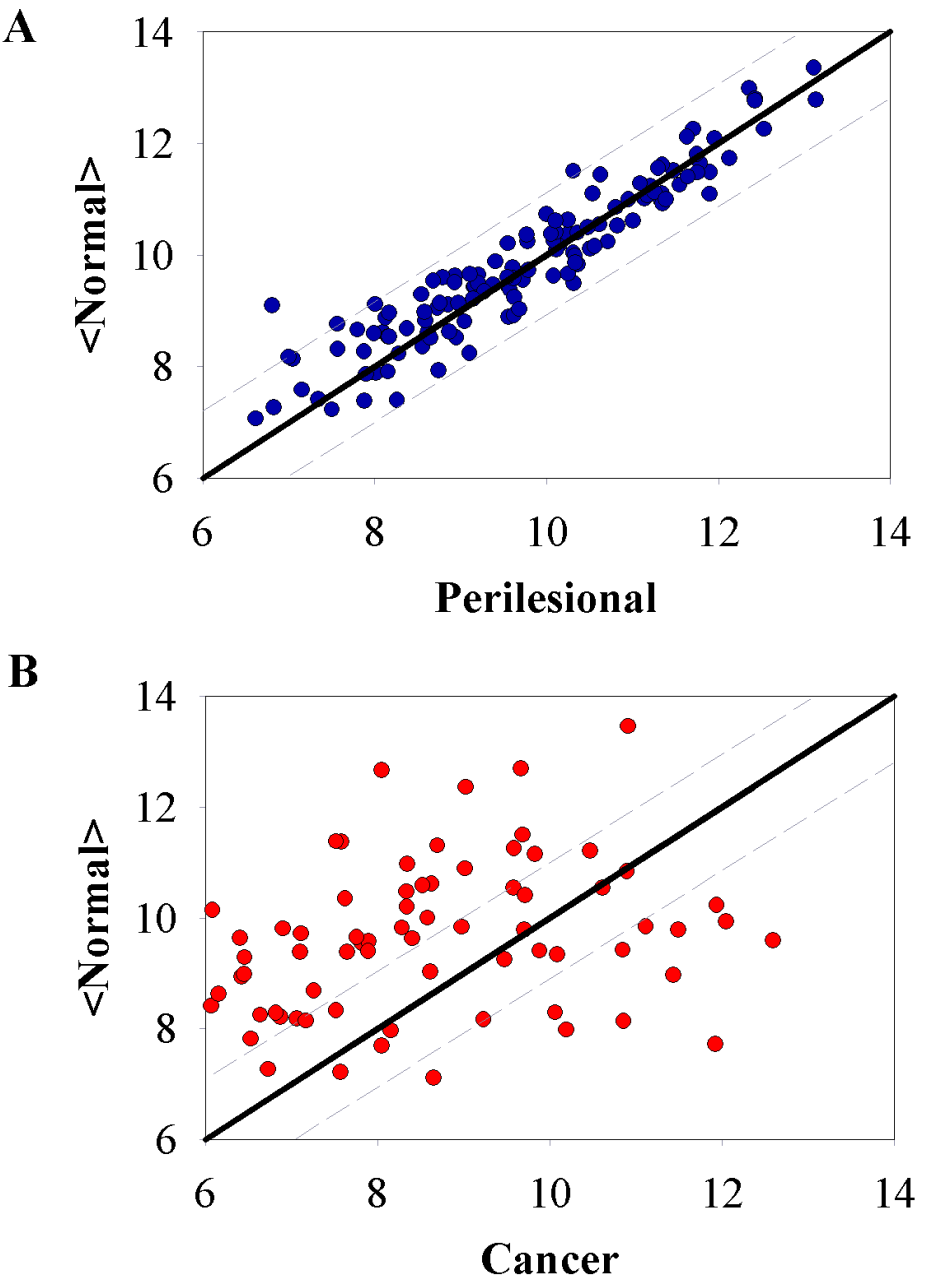
**Squamous cell carcinoma tumor and perilesional display distinctly different scatter plots from normal tissue**. Representative scatter plots of average normal versus perilesional (A) and cancer (B) samples from patient 1, for gene subset 1. R is depicted by the dashed line around the normal baseline line (slope = 1). The value for R is optimized for differentiation between groups for each subset of genes.

Approximate sample entropy analysis of microarray data has proven to be a reliable method of differentiating subgroups of patient samples. With the ability to classify the perilesional biopsy sample from normal with 75% accuracy, and to classify cancer with 80% accuracy with an 81 gene set (subset 1). (Table 1) The approximate sample entropy, which is a measurement of randomness in a system, was found to increase within a patient from perilesional to cancer with a 1.1 to 2.0 ratio. (Figure 5)

**Table 1:** Assessment of evaluation metrics for subset 1 and 2 from the logistic regression

**Training Data**	**Gene Subset 1**		
	
**Positive Category**	**Recall**	**Precision**	**F-Measure**
**Perilesional**	100.00%	100.00%	100.00%

**Cancer**	100.00%	100.00%	100.00%

**Validation Data**			

**Positive Category**	**Recall**	**Precision**	**F-Measure**

**Perilesional**	75.00%	75.00%	75.00%

**Cancer**	80.00%	80.00%	80.00%

**Training Data**	**Gene Subset 2**		
	
**Positive Category**	**Recall**	**Precision**	**F-Measure**

**Perilesional**	100.00%	85.71%	92.31%

**Cancer**	100.00%	100.00%	100.00%

**Validation Data**			

**Positive Category**	**Recall**	**Precision**	**F-Measure**

**Perilesional**	75.00%	75.00%	75.00%

**Cancer**	100.00%	83.33%	90.91%

Quantitative real-time validation has been performed with a limited gene set from the sample entropy analysis of the Affymetrix microarrays. Approximate sample entropy analysis of normalized quantitative real-time PCR data was also found to be reliable at differentiating subgroups of cervical neoplasia. Unlike the original gene array experimental set, in the validation study expanded the abnormal cervical sample groups to include CIN3 (the premalignant lesion), and stages I and II squamous cell cancer. We find in Table 2 that our classifications are correct in 85% of the population with gene subset 1, and for subset 2 the probability of accurately classifying a SCC patient with cancer is 94%. The probability distribution of cancer was found to be statistically different from the CIN3 distribution with p values less than 0.005 for both subsets. (Table 2)

**Table 2:** Probability distribution parameters and classification probabilities for the realtime validation of gene subsets 1 and 2

	**Subset 1**		**Subset 2**	
	
	**Cin3**	**SCC**	**Cin3**	**SCC**
	
**Mean**, m	0.424	1.122	0.539	1.87
	
**Standard Deviation**, s	0.176	0.455	0.304	0.699
	
**Probability of classifying Normal**	0.059757	0.016329	0.100342	0.006934
	
**Probability of classifying Cin3**	**0.850268**	0.138632	**0.704364**	0.05598
	
**Probability of classifying SCC**	0.089975	**0.845039**	0.195294	**0.937085**
	
**p value (T-test)**	0.0005		0.0001	

## Conclusion

**Figure 6:**
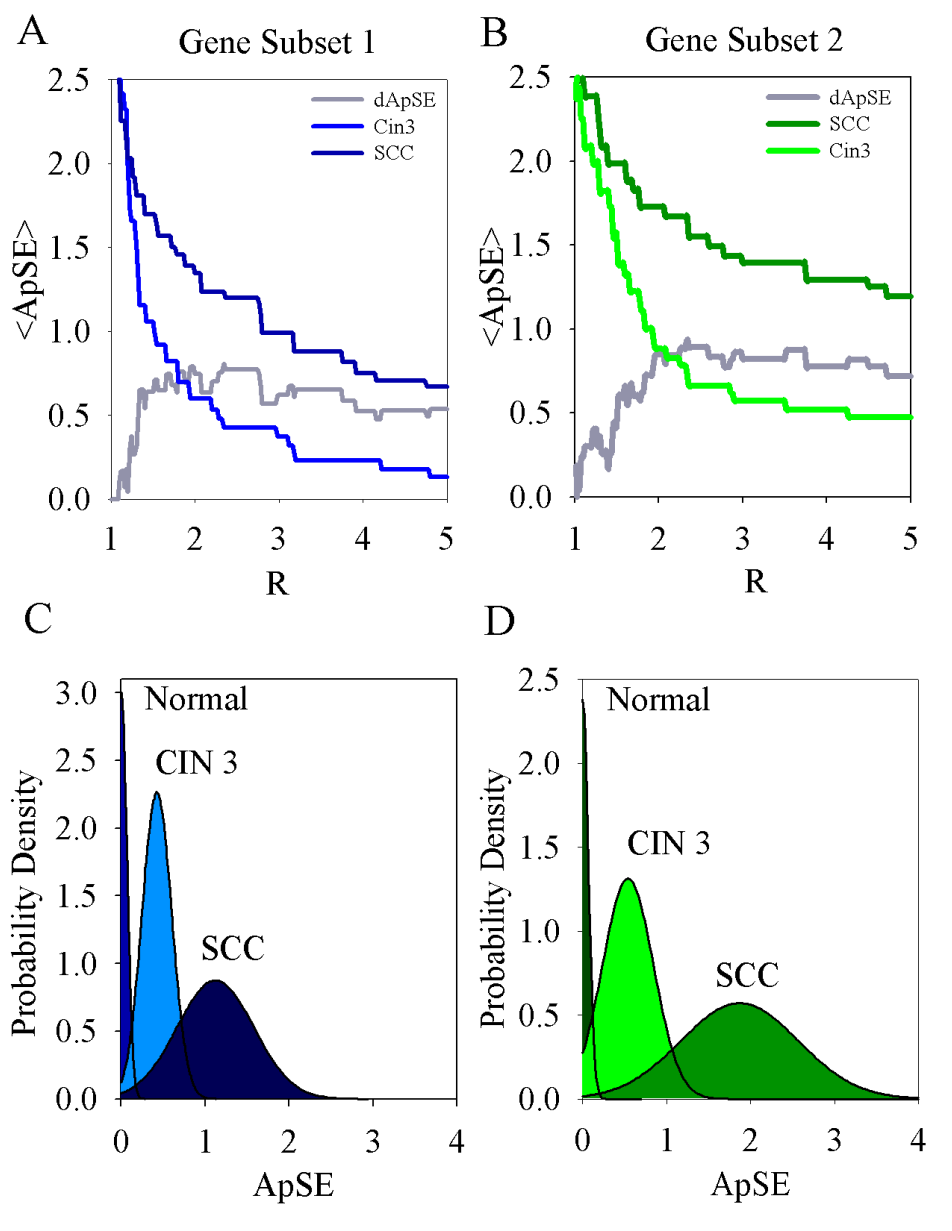
**Probability distribution of approximate sample entropy approach for gene sets 1 and 2 applied to an independent group of patients**. Plot of R versus average *ApSE *for SCC (dots, n = 11)(dark), CIN3(dashed, n = 7)(light), and d*ApSE *(*ApSE *[SCC]-*ApSE *[CIN3]) (grey), The value of R which results in the maximum change in *ApSE *is chosen to represent the gene subset 1 (A), and subset 2 (B). Normal probability distribution of approximate sample entropy calculated for Normal (n = 10), CIN 3 (n = 7) and SCC (n = 11) qRT-PCR validation study for subset 1 (C), and subset 2 (D).

The sample entropy analysis has the ability to detect abnormality with a predictive probability of 88%, with region R set at the maximum value for difference in *ApSE *between cancer and perilesional (*dApSE*). (Figure 6) The ability to accurately classify the cancer biopsy sample from the premalignant lesion with 81% accuracy, and to classify the malignant lesion with 48% accuracy. Noting that this gene set was determined by its ability to distinguish Stage I cervical cancer from normal cervix, it is encouraging to find the sample entropy values for CIN 3 distributed between normal and lower cancer values. CIN 3 lesions have varying prognosis, from spontaneous regression to 14% progression to invasive cancer. Our miss classification of 9–20% of CIN 3 and invasive cancer may reflect this subpopulation of CIN 3 with early invasive potential.

## Authors' contributions

SKB conceived of the study, participated in design, supervised the molecular studies and carried out the analysis. JBT assisted in development of analysis and draft of the manuscript. SAS carried out the molecular studies. MFB assisted in patient recruitment, sample acquisition and molecular studies. CDA participated in design and supervised all aspects of the study. All authors contributed to the writing of this manuscript. All authors read and approved the final manuscript.
